# The Microbiota in Long COVID

**DOI:** 10.3390/ijms25021330

**Published:** 2024-01-22

**Authors:** Carmen Álvarez-Santacruz, Sylwia D. Tyrkalska, Sergio Candel

**Affiliations:** 1 Servicio de Otorrinolaringología, Hospital de la Vega Lorenzo Guirao, 30530 Cieza, Spain; 2 Departamento de Biología Celular e Histología, Facultad de Biología, Universidad de Murcia, 30100 Murcia, Spain; tyrkalska.sylwia@gmail.com; 3 Instituto Murciano de Investigación Biosanitaria (IMIB)-Pascual Parrilla, 30120 Murcia, Spain; 4 Centro de Investigación Biomédica en Red de Enfermedades Raras (CIBERER), Instituto de Salud Carlos III, 28029 Madrid, Spain

**Keywords:** long COVID, SARS-CoV-2, COVID-19, microbiota, dysbiosis

## Abstract

Interest in the coronavirus disease 2019 (COVID-19) has progressively decreased lately, mainly due to the great effectivity of vaccines. Furthermore, no new severe acute respiratory syndrome coronavirus 2 (SARS-CoV-2) variants able to circumvent the protection of these vaccines, while presenting high transmissibility and/or lethality, have appeared. However, long COVID has emerged as a huge threat to human health and economy globally. The human microbiota plays an important role in health and disease, participating in the modulation of innate and adaptive immune responses. Thus, multiple studies have found that the nasopharyngeal microbiota is altered in COVID-19 patients, with these changes associated with the onset and/or severity of the disease. Nevertheless, although dysbiosis has also been reported in long COVID patients, mainly in the gut, little is known about the possible involvement of the microbiota in the development of this disease. Therefore, in this work, we aim to fill this gap in the knowledge by discussing and comparing the most relevant studies that have been published in this field up to this point. Hence, we discuss that the relevance of long COVID has probably been underestimated, and that the available data suggest that the microbiota could be playing a pivotal role on the pathogenesis of the disease. Further research to elucidate the involvement of the microbiota in long COVID will be essential to explore new therapeutic strategies based on manipulation of the microbiota.

## 1. Introduction

**Figure 1:**
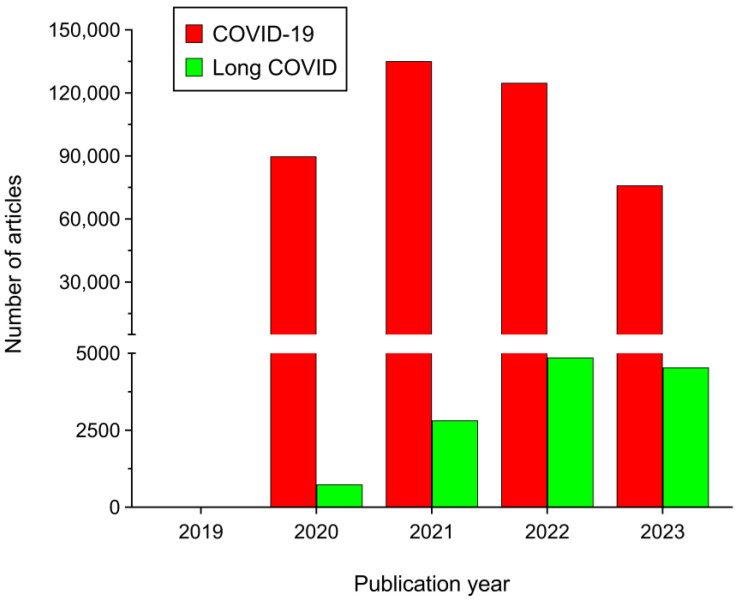
Number of items retrieved from PubMed database since the emergence of COVID-19 (between 2019 and 2023) searching for the terms ‘COVID-19′ (red bars) and ‘long COVID’ OR ‘post-COVID’ (green bars). Note that data from 2023 are up to 25 November 2023.

The novel betacoronavirus SARS-CoV-2 is the causative agent of COVID-19 [1]. Its sudden outbreak was declared as a global pandemic by the World Health Organization (WHO) on 11 March 2020 [2]. Fortunately, vaccines against COVID-19 have proven to be tremendously effective [3,4]. Moreover, there are no new SARS-CoV-2 variants emerging that are capable of circumventing the protection of such vaccines while presenting high transmissibility and/or lethality. These facts led the WHO to declare that COVID-19 was no longer a Public Health Emergency of International Concern (PHEIC) on 5 May 2023 [5]. All this has contributed to a progressive decrease in the levels of alarm and interest about the disease both in the scientific community and in the general population. Indeed, the number of scientific publications on COVID-19 began to decrease from 2022 (Figure 1). Nevertheless, like in a double-pan balance, while concern about COVID-19 progressively decreased, the chronic form of the disease (long COVID) was also gradually emerging as a potential major threat to human health globally. In fact, unlike with COVID-19, the number of scientific publications on long COVID has continued to increase every year (Figure 1). Thus, while COVID-19 symptoms disappear within the next few weeks after SARS-CoV-2 infection in most patients, they can persist in many COVID-19 convalescents, initiating the chronic phase of the disease. In the absence of any consensus, this chronic phase of COVID-19 has been termed as long COVID, post-acute sequelae of COVID-19 (PASC), post-COVID-19 condition, post-COVID-19 syndrome, chronic COVID syndrome (CCS), or post-acute COVID-19 syndrome (PACS) [6,7,8]. Several institutions have made efforts to establish a clear definition of long COVID that could be broadly accepted by the scientific community. Thus, the WHO proposed a clinical definition for long COVID in October 2021, stating that it generally occurs three months after the onset of COVID-19, with symptoms lasting at least two months and not explained by an alternative diagnosis [9]. Furthermore, the Centers for Disease Control and Prevention (CDC) defined the disease as a wide range of new, returning, or ongoing symptoms that people experience ≥4 weeks after being infected with SARS-CoV-2 [10]. Unfortunately, the efforts of both institutions, among others, were not very successful and the nomenclature, definition, prevalence, epidemiology, pathogenesis, and mechanism of long COVID are still unclear.

The human microbiota is the set of ecological communities of microorganisms present inside and on the surface of our body, including bacteria, archaea, fungi, viruses, and protists [11,12]. It plays a pivotal role in health and disease [13,14]. Importantly, numerous studies have found unquestionable correlations between microbiota composition and the susceptibility of individuals to multiple viral infections, including COVID-19 [15,16]. The gut microbiota has always been the most studied among all the different human anatomical areas where the microbiota is present [16,17]. This is due to the fact that the gut has long been considered as the main location of the human microbiota and the one that harbours the largest collection of microorganisms by far [18]. Therefore, the field of long COVID has not been an exception and most studies analysing the possible role of the microbiota in this disease have also focused on the gut microbiota. Furthermore, this has also been motivated by the finding that it has already been amply demonstrated that the gut microbiota composition is altered in patients with COVID-19 [19,20]. The upper respiratory tract is key for initial SARS-CoV-2 infection and proliferation, especially the nasopharynx, which is the common meeting place for the main portals of entry for viruses: ear, nose, and oral cavities [21]. In fact, the nasopharynx presents higher viral loads than any other anatomical area in SARS-CoV-2-infected subjects [22,23]. This has made nasopharyngeal swabs the “gold standard” for the diagnosis of this infection [23]. In addition, many recent studies have demonstrated that changes in the nasopharyngeal microbiota correlate with increased or reduced susceptibility to different viral infections in humans [15]. All this has led scientists to perform dozens of studies analysing the nasopharyngeal microbiota of COVID-19 patients [16]. Surprisingly, these studies have reported highly variable and contradictory results, and the potential sources of such variability were analysed and discussed in depth in a recent review article [16]. Therefore, here, we are not addressing general aspects such as the definition of the different terms used in the field of metagenomics, nor the differences between the distinct sequencing technologies, nor human microbiota diversity, anatomical distribution, abundance, or its role in health and disease, since we already addressed all this in the aforementioned review article [16].

Several studies have already found that the microbiota of different anatomical areas is altered in long COVID patients, suggesting the possible existence of links between dysbiosis and susceptibility to and/or severity of long COVID. These are the results we intend to analyse and discuss herein. However, the panorama on this topic is very different from that found when we analysed the nasopharyngeal microbiota in COVID-19. Thus, in this case, there are still many fewer studies, but with interesting findings that have not yet been compared and discussed together in an integrated manner as we review here, hence the need for, and interest of, this work.

## 2. COVID-19 and Long COVID: Two Different Sides of the Same Coin

The diagnosis of COVID-19 has become easy, fast, and totally standardized, thanks to the existence of reliable techniques that detect viral RNA by RT-PCR or viral antigens (Table 1) [24,25]. On the contrary, the lack of consensus on the definition of long COVID, and the different criteria used by clinicians to diagnose it, hinders its study and knowledge. For example, the incidence of long COVID is difficult to estimate due to this vagueness, with studies showing percentages of SARS-CoV-2 infected individuals with long COVID symptoms that range from 10% to over 70% (Table 1) [26,27]. Furthermore, the most common long COVID symptoms are difficult to differentiate from those of a normal, prolonged convalescence, or of other post-infection syndromes triggered by infection and immune activation, such as post-viral fatigue (Table 1) [28]. This makes long COVID a diagnosis of exclusion in many cases [29]. Hundreds of symptoms across multiple organ systems have been described in both COVID-19 and long COVID (Table 1) [30,31]. Many of them are common, albeit long COVID is characterized by the appearance of new-onset conditions such as type 2 diabetes [32], cardiovascular, thrombotic, and cerebrovascular disease [33], and myalgic encephalomyelitis/chronic fatigue syndrome (ME/CFS) (Table 1) [34]. COVID-19 symptoms usually disappear within the following few weeks after infection in a significant proportion of subjects [30], whereas long COVID manifestations can last for years [31], especially in cases of ME/CFS which are expected to be lifelong (Table 1) [35].

The most important risk factors for COVID-19 and long COVID have already been identified (Table 1) [36,37]. Individuals who were hospitalized or needed ICU care during the acute phase of COVID-19 present more than twice the risk of developing long COVID (Table 1) [37]. However, surprisingly, no association has been found between the severity of both diseases [36,37]. In any case, recent advances in the knowledge of the pathogenesis of COVID-19 suggest that infection of the lower respiratory tract by SARS-CoV-2 results in alveolar damage due to dysfunctional immune responses (Table 1) [38]. This leads to increased epithelium and endothelium permeability that promotes inflammation and coagulation, while the influx of immune cells induces exacerbated inflammatory responses and immunopathology (Table 1) [38]. Nevertheless, the pathogenesis of long COVID remains a conundrum, with several potentially overlapping hypothesized mechanisms, including immune dysregulation, autoimmunity, clotting and endothelial abnormality, dysfunctional neurological signalling, and microbiota disruption (Table 1) [6].

Unlike COVID-19, for which there are effective preventive measures [3,4] and treatments [39], the prevention and treatment of long COVID are not so well established. Since suffering from severe COVID-19 doubles the risk of developing long COVID [37], all the strategies that prevent the infection by SARS-CoV-2 or reduce COVID-19 disease severity could also be preventing the development of long COVID (Table 1). There are currently no effective treatments for long COVID, and the different symptoms are treated individually (Table 1) [6].

The microbiota alterations observed in long COVID patients, which have led to the mentioned hypothesis suggesting that dysbiosis may be playing a paramount role in the pathogenesis of the disease (Table 1), will be the main topic discussed in this work.

**Table 1:** Comparison between COVID-19 and long COVID main features.

	COVID-19	Long COVID
Timelines	Symptoms usually appear between days 2 and 14 after infection by the SARS-CoV-2 virus [30]	It varies depending on the different definitions: according to the World Health Organization it occurs 3 months after the onset of COVID-19, with symptoms lasting at least 2 months and not explained by an alternative diagnosis [9]; according to the Centers for Disease Control and Prevention it occurs when a wide range of new, returning, or ongoing symptoms are present 4 weeks after SARS-CoV-2 infection [10] According to the onset of the different symptoms, parosmia appears 3 months after infection [40]; paraesthesia, hair loss, blurry vision, and swelling of the legs, hands, and feet are more common at 2 months after infection [41]; pain in joints, bones, ears, neck, and back are more common at 1 year after infection [41]; neurocognitive symptoms worsen over time and tend to persist longer [31,42]; gastrointestinal and respiratory symptoms are the most likely to resolve [31,42]
Diagnosis	Easy, rapid, cheap, and reliable, thanks to the availability of diagnostic tools that detect viral RNA (RT-qPCR) [24] or viral antigens [25]	Unclear, due to the lack of consensus about the definition of long COVID, the different diagnostic criteria used by distinct clinicians, and the similarity between its symptoms and those from other post-infection syndromes such as post-viral fatigue [28]. Blood tests, checking blood pressure and heart rate, chest X-ray, and measuring oxygen levels are the diagnostic tests commonly used, but long COVID usually is a diagnosis of exclusion [29]
Incidence	772,166,517 cumulative cases worldwide to date (https://covid19.who.int/, accessed on 22 November 2023)	10–30% of non-hospitalized cases [26,27] 50–70% of hospitalized cases [26,27] 10–12% of vaccinated cases [43,44]
Most frequent symptoms	Systemic symptoms: elevated temperature, exhaustion, and weakness Cardiovascular symptoms: palpitations, acute myocardial injury, acute pericarditis, blood pressure abnormalities, and myocarditis Ophthalmic symptoms: conjunctivitis, anterior uveitis, retinitis, and optic neuritis Musculoskeletal symptoms: body ache and arthralgia Respiratory symptoms: runny nose, cough, sore throat, and shortness of breath Gastrointestinal symptoms: diarrhoea, loss of appetite, nausea, vomiting, anorexia, and abdominal pain Olfactory and gustatory symptoms: hyposmia, anosmia, hypogeusia, and ageusia Neurological symptoms: headache, confusion, loss of speech, languidness, and malaise Dermatologic symptoms: erythematous rash, widespread urticaria, chickenpox-like vesicles, urticarial eruptions, and petechiae [30]	Systemic symptoms: fatigue, post exertional malaise, elevated temperature, chills, and skin sensations Reproductive, genitourinary, and endocrine symptoms: extreme thirst, all menstrual/period issues, abnormally irregular periods, abnormally heavy periods, and sexual dysfunction Cardiovascular symptoms: palpitations, tachycardia, pain/burning in chest, visibly inflamed/bulging veins, and bradycardia Musculoskeletal symptoms: tightness of chest, muscle aches, joint pain, stiff neck, and muscle spasms Immunologic and autoimmune symptoms: heightened reaction to old allergies, new allergies, new anaphylaxis reaction, and shingles Neurological symptoms: brain fog, headache, loss of speech, and confusion Respiratory symptoms: shortness of breath, dry cough, breathing difficulty (normal O2 level), cough with mucus production, and sneezing Gastrointestinal symptoms: diarrhoea, loss of appetite, nausea, abdominal pain, and gastroesophageal reflux Dermatologic symptoms: itchy skin, skin rashes, petechiae, COVID toe, and peeling skin [31]
Pathogenesis	Dysfunctional immune responses to the infection of the lower respiratory tract by the SARS-CoV-2 virus damage the pulmonary alveoli. This leads to an increase in epithelium and endothelium permeability, which promotes inflammation, coagulation, and the influx of immune cells that induce exacerbated inflammatory responses and immunopathology [38]	Several, potentially overlapping, hypothesis, highlighting [6]: – Persisting reservoirs of SARS-CoV-2 in tissues [45] – Immune dysregulation with or without reactivation of underlying pathogens, including herpesviruses such as Epstein–Barr virus (EBV) and human herpesvirus 6 (HHV-6) among others [46,47] – Impacts of SARS-CoV-2 on the microbiota [19,48] – Autoimmunity and priming of the immune system from molecular mimicry [49,50,51] – Microvascular blood clotting with endothelial dysfunction [51] – Dysfunctional signalling in the brainstem and/or vagus nerve [52]
Risk factors	Higher age, male sex, post-menopausality, higher body mass index, smoking, longer waiting time to admission, and preexisting comorbidities (including hypertension, cardiovascular disease, diabetes cerebrovascular disease, tuberculosis, chronic renal disease, and chronic obstructive pulmonary disease) [36]	Female sex, older age, higher body mass index, smoking, and preexisting comorbidities (including anxiety and/or depression, asthma, COPD, diabetes, IHD, and immunosuppression). Moreover, patients who needed hospitalization or ICU care during the acute phase of COVID-19 present more than twice the risk of developing long COVID [37]
Prevention and treatment	Prevention: social distancing and prophylactic measures to avoid infection by SARS-CoV-2, and vaccines Treatment: – Antiviral agents: Remdesivir, Favipiravir, Ribavirin, interferons, Ritonavir, Lopinavir, Arbidol, Chloroquine/Hydroxychloroquine, recombinant soluble ACE2, Azithromycin, Ivermectin, Nitazoxanide, Camostat mesylate, and Paxlovid – Biologic agents: monoclonal antibodies, convalescent plasma, hyperimmune sera, and exogenous surfactant delivery – Anti-inflammatory agents: corticosteroids, Fluvoxamine, Anakinra, granulocyte-macrophage colony-stimulating factor (GM-CSF) inhibitors, intravenous immunoglobulin, Janus kinase (JAK) inhibitors, and colchicine – Traditional Chinese medicine herbal agents: Atractylodis Macrocephalae Rhizoma (Baizhu), Fructus forsythia (Lianqiao), Lonicerae Japonicae Flos, Saposhnikoviae Radix (Fangfeng), Glycyrrhizae Radix Et Rhizoma (Gancao), and Astragali Radix (Huangqi) [39]	Prevention: all the prevention and treatment strategies that reduce risk of suffering from severe COVID-19 Treatment: there are currently no effective treatments for long COVID, but the different symptoms are treated individually, including pacing to treat postexertional malaise, probiotics and/or diet changes to treat gastrointestinal symptoms, apheresis and anticoagulants to treat abnormal clotting, naltrexone to treat pain and fatigue, or intravenous immunoglobulin to treat immune dysfunction [6] Notably, nutritional supplements such as the combination of hydroxytyrosol, acetyl L-carnitine, and vitamins B, C, and D [53], the combination of L-Arginine and Vitamin C [54], Apportal^®^ [55], or Requpero^®^ [56], have been shown to improve long COVID symptoms Moreover, Sulodexide significantly improves long-lasting post-COVID-19 endothelial dysfunction and alleviates chest pain and palpitations [57]

## 3. The Gut Microbiota in Long COVID

As in the majority of the diseases for which pathogenesis has been related to dysbiosis, possible alterations of the gut microbiota have also been the most studied in patients with long COVID [16,17]. This is motivated by the fact that the gut microbiota is considered the most relevant microbiota in health and disease [16,18], but also because multiple studies have shown that it is significantly altered in COVID-19 patients [19,20]. The choice of the timepoints when the microbiota is analysed is a crucial factor for works studying whether SARS-CoV-2 infection can induce persistent changes in the gut microbiota that may be involved in the onset and/or severity of long COVID. Regarding this, there is a wide variety of experimental designs among the studies that have addressed this topic. Thus, we will begin our analysis with the works that have studied shorter times and will continue in increasing order. Shortly after the beginning of the COVID-19 pandemic, the study conducted by Zuo and colleagues already showed that opportunistic pathogens were overrepresented whilst beneficial commensals were underrepresented in the gut of COVID-19 patients (Table 2) [20]. Moreover, they found several associations between changes in the abundance of certain bacterial taxa and the severity of COVID-19 [20]. Curiously, this gut dysbiosis was already present at the time of hospitalization, was detected at all timepoints during hospitalization, and persisted even after the clearance of SARS-CoV-2 and the resolution of the respiratory symptoms (Table 2) [20]. Tian and colleagues used 16S rRNA gene sequencing to compare the gut microbiota of seven uninfected controls and seven COVID-19-recovered patients at three months after discharge (Table 2) [58]. Coherently, they observed that alpha and beta diversity values were significantly different when comparing both groups (Table 2) [58]. Another work performed by Upadhyay and colleagues analysed the gut microbiota at later timepoints after SARS-CoV-2 initial infection (up to 154 days) (Table 2) [59]. Notably, this study could give us some clues about why no association has been found between long COVID onset or severity and the severity of the acute COVID-19 disease, thus finding many severely ill long COVID patients with a history of mild or even asymptomatic COVID-19 (Table 2) [60,61]. On the one hand, these authors demonstrated that SARS-CoV-2 altered the gut microbiota not only in the K18-humanized angiotensin-converting enzyme 2 mouse model, which is susceptible to SARS-CoV-2 infection, but also in wild-type C57BL/6J mice that are resistant to severe lung pathology from SARS-CoV-2 infection [59]. On the other hand, they found that mild SARS-CoV-2 infection resulted in long-lasting disruption and instability of the gut microbial ecology, with the Firmicutes and Actinobacteriota phyla and the *Rothia* genus as the most variable bacterial taxa following SARS-CoV-2 infection (Table 2) [59]. For this, they used 16S rRNA gene sequencing and shotgun metagenomic sequencing (MGS) to evaluate the gut microbiota in stool samples of SARS-CoV-2-positive human patients with mild symptoms at different times after initial infection (Table 2) [59]. Taken together, these results suggest that the long-lasting gut dysbiosis resulting from SARS-CoV-2 infection, which could be later playing a pivotal role on the pathogenesis of long COVID, is independent on the severity of the acute phase of the COVID-19 disease. It is worth highlighting that the vast majority of studies on long COVID enrol COVID-19 patients with a wide spectrum of disease severities, without stratifying them by severity in most cases (Table 2). This is to avoid any possible biases that could arise if a given COVID-19 disease severity is over- or under-represented. However, the work performed by Upadhyay and colleagues is particularly useful as they exclusively focused on mild COVID-19 patients [59]. Hence, they obtained valuable information regarding the possible associations between the severity of COVID-19 and long-lasting changes in the gut microbiota, which could lead to the onset of long COVID [59]. Unfortunately, although interesting, these three already-mentioned studies presented very low sample sizes as their main limitation, which could compromise the soundness of their conclusions (Table 2) [20,58,59]. Moreover, none of the three followed the evolution of the patients to know which ones developed long COVID to compare them with those others who did not develop the disease (Table 2) [20,58,59].

Interestingly, Chen and colleagues went one step further in terms of the time elapsed from the initial infection to the time of analysis (Table 2) [62]. Thus, they studied the gut microbiota at three different timepoints after the SARS-CoV-2 infection, the longest being 6 months after discharge (Table 2) [62]. Specifically, they utilised 16S rRNA gene sequencing to monitor alterations in the faecal microbiota of COVID-19 patients with diverse disease severity at three timepoints: (1) acute phase, from illness onset to viral clearance; (2) convalescence, from viral clearance to 2 weeks after hospital discharge; and (3) postconvalescence, 6 months after hospital discharge (Table 2) [62]. Importantly, their data also supported the idea that SARS-CoV-2 infection results in a long-lasting disruption of the gut microbiota [59], since they found that bacterial alpha diversity was significantly reduced in acute phase, convalescence, and postconvalescence patients to the same levels compared to uninfected controls (Table 2) [62]. However, their sample sizes were also low, they did not perform any relative abundance analyses, and the potential correlations between changes in alpha diversity and the onset or severity of long COVID were not explored (Table 2) [62]. As in the previous work, Liu and colleagues also extended their gut microbiota analyses up to 6 months after initial SARS-CoV-2 infection (Table 2) [48]. However, unlike the four studies already mentioned, in this case they established a group of long COVID patients (Table 2) [48]. These authors used MGS to analyse changes in faecal samples of 106 COVID-19 patients with different disease severity from admission until 6 months later, and then correlated the results with persistent symptoms at 6 months (Table 2) [48]. They observed that the gut microbiota of COVID-19 patients who did not develop long COVID was totally recovered and indistinguishable from that of uninfected controls at 6 months (Table 2) [48]. Nevertheless, the 76% of patients who developed long COVID presented significant compositional alterations of gut microbiota at the same time after infection (Table 2) [48]. Importantly, their analyses revealed significant correlations between the abundance of certain bacterial taxa and the presence of some of the most characteristic symptoms of long COVID (Table 2) [48]. Hence, the abundance of nosocomial gut pathogens, including *Clostridium innocuum*, was high in patients with fatigue and neuropsychiatric symptoms, whereas the abundance of opportunistic gut pathogens was high in patients with persistent respiratory symptoms (Table 2) [48]. This study avoided the main limitations of the other works mentioned before, as sample sizes were reasonably high, and samples were homogeneously collected at 6 months after initial infection (Table 2) [48]. However, the COVID-19 disease severity of their enrolled patients was not taken into account, losing the opportunity to perform additional interesting comparisons, especially after having found that the gut microbiota composition at admission was associated with the occurrence of long COVID (Table 2) [48]. At this point, an apparent contradiction arises between the finding that the gut microbiota was restored in COVID-19 patients who did not develop long COVID observed by Liu and colleagues [48], and the long-lasting gut dysbiosis in all COVID-19 patients described in the previously discussed works by Tian and colleagues [58], Upadhyay and colleagues [59], and Chen and colleagues (Table 2) [62]. Nevertheless, these differences are probably because, while Liu and colleagues separated COVID-19 patients who developed long COVID and those who did not into two different groups (Table 2) [48], all COVID-19 patients were analysed together in the other three studies (Table 2) [58,59,62]. Therefore, in these three studies the long-lasting gut dysbiosis observed in patients who developed long COVID could be concealing the recovery of gut microbiota in patients who did not develop long COVID (Table 2) [58,59,62]. To elucidate this issue, Zhang and colleagues published what probably is the most clarifying study on this topic to date, mainly because their sample sizes were very high, and they extended the time at which the gut microbiota was analysed up to 1 year after discharge (Table 2) [63]. They performed 16S gene sequencing of stool samples from three different groups of subjects at 1 year after discharge: (1) healthy controls; (2) COVID-19-recovered patients without persistent symptoms; and (3) COVID-19 patients who presented long COVID symptoms (Table 2) [63]. Then, they analysed the correlations between the gut microbiota and long COVID, finding that, consistent with the results from Chen and colleagues [62], alpha diversity was significantly reduced in long COVID patients compared to both control groups (Table 2) [63]. However, while Chen and colleagues observed that alpha diversity decreased in all their groups of subjects up to 6 months after discharge compared to uninfected controls [62], Zhang and colleagues found that alpha diversity was normal in both uninfected controls and COVID-19 patients who did not develop long COVID at 1 year after discharge (Table 2) [63]. Thus, this sheds light on the previous discussion and reinforces the idea that the gut microbiota of COVID-19 patients who did not develop long COVID has probably been restored at 6 months after initial infection as shown by Liu and colleagues [48], and at 1 year after discharge as shown by Zhang and colleagues [63]. In any case, besides these changes in alpha diversity, Zhang and colleagues also analysed the compositional structure of the gut microbiota (Table 2) [63]. They observed that the relative abundance of the bacterial genera *Eubacterium*, *Agathobacter*, *Subdoligranulum*, and *Ruminococcus* was significantly different in COVID-19 patients who developed long COVID compared to both control groups, whereas, on the contrary, the genus *Veillonella* was overrepresented in long COVID patients (Table 2) [63].

In conclusion, taken together, all these results strongly suggest that SARS-CoV-2 infection induces long-lasting gut dysbiosis in COVID-19-recovered patients who develop long COVID. However, the gut microbiota of COVID-19 patients who do not develop long COVID is already restored at 6 months after the initial SARS-CoV-2 infection, or probably even earlier, although further research would be necessary to determine this. Therefore, new studies analysing multiple timepoints after the SARS-CoV-2 initial infection will be necessary to characterize the exact dynamics of changes in the gut microbiota of patients in recovery, as well as to elucidate the involvement of gut dysbiosis in the pathogenesis of long COVID.

## 4. The Upper Respiratory Tract Microbiota in Long COVID

The upper respiratory tract, and especially the nasopharynx, has been shown to be key for SARS-CoV-2 infection and proliferation [16,21]. The nasopharynx presents a common meeting place for the ear, nose, and oral cavities, which are the main portals of entry for the virus [16,21]. Thus, the nose and oral cavities are particularly important for the entry of respiratory viruses, including SARS-CoV-2, being the first places where their replication starts [16,21]. In the human body, only the gut microbiota is larger than that of the mouth, whose microbial community comprises over 1000 species of commensal bacteria, viruses, fungi, and protozoa [64]. Microorganisms from the oral cavity can be aspirated or ingested, altering the microbiota of the new anatomical areas to which they reach and potentially producing diseases such as pneumonia [65,66]. Importantly, oral dysbiosis has been linked to the pathogenesis of multiple systemic diseases [67,68]. Undoubtedly, the most important contribution to date to the knowledge of the relationship between the oral microbiota and long COVID was the work published by Haran and colleagues [69]. They collected tongue swabs from SARS-CoV-2-infected patients presenting COVID-19 symptoms and followed their evolution, finding that 37% of them developed long COVID [69]. Importantly, they found that the oral microbiota of long COVID patients presented higher abundance of the bacterial genera *Prevotella* and *Veillonella* [69]. Interestingly, it has been described that these bacterial genera can reach and infect the lungs through the oral–lung aspiration axis [70,71], and have been linked to systemic diseases [72]. *Prevotella* and *Veillonella* induce inflammation by TLR-2 activation and induction of the cytokines IL-23 and IL-1 in the case of *Prevotella* [73,74], and mainly by a strong induction of IL-6 in the case of *Veillonella* [75]. Notably, the low-grade inflammation induced by members of the *Prevotella* genus is known to be systemic [72]. Moreover, authors found increased abundance of lipopolysaccharide-producing bacterial species in samples from long COVID patients compared to controls, including *Veillonella dispar*, *Veillonella infantium*, *Veillonella atypica*, *Leptotrichia wadei*, and *Megasphaera micronuciformis* [69,76,77,78]. Another interesting observation is that metabolic pathways known to have anti-inflammatory properties were reduced [69]. Therefore, all these findings suggest that the inflammation induced by some of the bacterial genera that were overrepresented in the oral cavity of long COVID patients could be playing a pivotal role in the origin of their long-lasting symptoms. This is consistent with the widespread hypothesis that systemic chronic inflammation may be involved in the pathogenesis of long COVID [79,80]. One of the main strengths of this study was that authors collected samples at initial COVID-19 stages when patients had not received any treatment that could alter their microbiota [69]. In addition, they obtained reliable control samples from patients who did not develop long COVID but that were collected exactly in the same conditions and at the same timepoints as the long COVID samples [69]. However, sample sizes were low (with only 10 long COVID patients), and the study was devoid of another control group composed of uninfected subjects which would have allowed for additional interesting comparisons [69].

In summary, although the involvement of the upper respiratory tract microbiota in the onset and/or severity of long COVID has barely been studied yet, there are already important findings describing the existence of oral dysbiosis in long COVID patients [69]. As it has been amply demonstrated that the microbiota of other parts of the upper respiratory tract, especially that of the nasopharynx, is significantly altered in COVID-19 patients [16], further research will be necessary to determine whether it plays a role in the pathogenesis of long COVID.

## 5. Final Considerations and Conclusions

The lack of consensus about the clinical criteria to diagnose long COVID, together with the fact that its symptoms are usually difficult to distinguish from those of other post-infection syndromes, hinders the knowledge of this disease, including its incidence. Unfortunately, all this together could be contributing to underestimating the relevance and incidence of long COVID, making it more difficult to raise awareness among the general population and funding bodies about the urgent need to dedicate more efforts to research on this topic. Therefore, with significant proportions of individuals suffering from disabling long COVID symptoms and even being unable to return to work [31], establishing clear and standardized diagnostic criteria would be essential to give this disease the relevance it deserves. This would help advance the study of the mechanisms underlying long COVID pathogenesis and the development of novel therapeutic strategies.

SARS-CoV-2 infection can induce long-lasting gut dysbiosis in both mice that are resistant to severe lung pathology and COVID-19 patients with mild symptoms. These results suggest that the gut microbiota could be involved in the intriguing and alarming observation that a multitude of severely ill long COVID patients had a mild or even asymptomatic COVID-19. Furthermore, different studies that analyse the persistency of gut dysbiosis in COVID-19 patients and/or subjects with long COVID suggest that SARS-CoV-2 infection induces long-lasting gut dysbiosis in COVID-19 patients who develop long COVID. However, the gut microbiota of COVID-19 patients who do not develop long COVID is restored at 6 months after the initial infection. New studies analysing the gut microbiota of COVID-19 patients in recovery at different timepoints will be necessary to characterize the dynamics of this recovery, and to study why such dynamics are different in the subjects who develop long COVID.

Little is known about the upper respiratory tract microbiota in long COVID, although its alterations in COVID-19 patients have been extensively studied, including the oral, nasal, oropharyngeal, and mainly the nasopharyngeal microbiotas. In addition, in many cases, this upper respiratory tract dysbiosis persists even after both the SARS-CoV-2 virus and COVID-19 symptoms have disappeared. Notably, the relevant bacterial abundance changes identified in the oral microbiota of long COVID patients that could be inducing persistent inflammation reinforce the idea that the microbiota of the upper respiratory tract may be playing a pivotal role in the pathogenesis of long COVID. Therefore, all this together suggests that characterizing the respiratory microbiota of long COVID patients and its possible involvement in the pathogenesis of the disease will deserve further research.

Interestingly, it has been demonstrated that distinct SARS-CoV-2 variants differentially alter the gut microbiota in mice [59], whilst a recent systematic review and meta-analysis showed that several long COVID symptoms vary depending on the SARS-CoV-2 variant that initiated the infection [81]. This could be interpreted as another piece of evidence suggesting a possible role of the microbiota on the onset and/or severity of long COVID, pointing to the SARS-CoV-2 variant that caused the initial infection as another factor to take into account in future investigations. In addition, further research will be necessary to determine whether dysbiosis could result in the release of bacterial toxins, which may alter the function of the mitochondria, thus contributing to the fatigue present in long COVID patients [82]. Finally, and even more importantly, it is worth mentioning that the virome, mycobiome, metabolome, and metatranscriptome of long COVID patients have not been studied in depth to date, being other factors that could be important as well and, therefore, require further research.

Strikingly, faecal transplantation from post-COVID patients is sufficient to induce alterations that resemble the symptoms of the disease in germ-free mice, including lung inflammation, worse outcomes during pulmonary infection, and poor cognitive performance [83]. This strongly suggests that the gut microbiota can directly contribute to long COVID sequelae, and that it may be a potential therapeutic target. There are already some promising results on this direction, highlighting the case of SIM01, which is an oral, microencapsulated formulation of three lyophilized species of the *Bifidobacterium* genus and three prebiotics known to be beneficial for the growth of these bacteria, offering a total of 20 billion colony-forming units per daily dose [84,85]. This oral microbiome formula is able to reduce adverse health outcomes of COVID-19 in elderly patients and patients with type 2 diabetes, hastens antibody formation against SARS-CoV-2, reduces nasopharyngeal viral load and pro-inflammatory immune markers, and restores gut dysbiosis in COVID-19-hospitalized patients [84,85]. It would be interesting to analyse the results of providing SIM01 to long COVID patients, which is the next logical step.

In conclusion, the great, and probably underestimated, relevance of long COVID and its huge impact on global health and economy, and the multiple pieces of evidence discussed here suggesting that dysbiosis could be playing a pivotal role on the pathogenesis of the disease, are ample reasons to encourage research on this topic with the aim of opening novel therapeutic avenues based on manipulation of the microbiota.

## Figures and Tables

**Table 2:** Research articles reporting changes in the gut microbiota that are discussed in this work, and summary of their most relevant findings. Research articles have been displayed in chronological order according to their publication date, to facilitate the understanding of how knowledge in the field has evolved over time. Note that, to avoid confusion, only results obtained by analysing human samples have been included here.

Reference	Publication Date	Sample Size and Relevant Features	Methodology	Timing of Analysis	Main Findings
[20]	September 2020	N = 36 (15 COVID-19 hospitalized patients, 6 subjects with community acquired pneumonia, and 15 uninfected controls) Relevant features: COVID-19 patients were stratified by disease severity (mild, moderate, severe, or critical)	Metagenomic sequencing Sequencing platform: Illumina NextSeq 550 (Illumina, San Diego, CA, USA)	Stool samples were collected two or three times per week from time of hospitalization until discharge	Opportunistic pathogens were overrepresented and beneficial commensals were underrepresented in COVID-19 patients at time of hospitalization and at all timepoints during hospitalization Gut dysbiosis persisted even after the clearance of SARS-CoV-2 and the resolution of the respiratory symptoms While the abundance of *Coprobacillus*, *Clostridium ramosum*, and *Clostridium hathewayi* correlated with COVID-19 severity, the correlation between the abundance of *Faecalibacterium prausnitzii* (an anti-inflammatory bacterium) and disease severity was inverse
[58]	May 2021	N = 14 (7 COVID-19-recovered patients and 7 uninfected controls) Relevant features: all the enrolled subjects were males	16S rRNA gene sequencing (V3–V4) Sequencing platform: Illumina MiSeq (Illumina, San Diego, CA, USA)	An average of 3 months after discharge	Alpha and beta diversity values were significantly different in COVID-19-recovered patients compared to uninfected controls at 3 months after discharge
[62]	January 2022	N = 60 (30 COVID-19 patients and 30 uninfected controls) Relevant features: Samples from COVID-19 patients with different disease severity were analysed at different timepoints: (1) acute phase, from illness onset to viral clearance; (2) convalescence, from viral clearance to 2 weeks after hospital discharge; and (3) postconvalescence, 6 months after hospital discharge	16S rRNA gene sequencing (V3–V4) Sequencing platform: Illumina MiSeq (Illumina, CA, USA)	Stool samples were analysed at three different timepoints, according to the groups of patients established, up to 6 months after discharge	Alpha diversity significantly decreased in acute phase, convalescence, and postconvalescence patients to the same levels compared to uninfected controls
[48]	March 2022	N = 106 (COVID-19 patients with different disease severity) Relevant features: 68 (76%) of the patients had developed long COVID at 6 months, whereas the remaining 38 (24%) patients had not	Metagenomic sequencing Sequencing platform: Illumina NextSeq 550 (Illumina, CA, USA)	Follow-up of patients since the diagnosis of COVID-19 up to 6 months	Gut microbiota composition at admission was associated with the occurrence of long COVID The gut microbiota of COVID-19 patients who did not develop long COVID was totally recovered and indistinguishable from that of uninfected controls at 6 months Patients who developed long COVID presented significant compositional alterations of gut microbiota at the same time after infection Their analyses revealed significant correlations between the abundance of certain bacterial taxa and the presence of some of the most characteristic symptoms of long COVID, such as the high abundance of nosocomial gut pathogens, including *Clostridium innocuum*, in patients with fatigue and neuropsychiatric symptoms, or the high abundance of opportunistic gut pathogens in patients with persistent respiratory symptoms
[63]	April 2023	N = 219 (187 COVID-19-recovered patients and 32 uninfected controls) Relevant features: 84 (44.9%) of the long COVID-19-recovered patients developed long COVID at 1 year after discharge	16S rRNA gene sequencing (V3–V4) Sequencing platform: Illumina MiSeq PE300 platform/NovaSeq PE250 platform (Illumina, CA, USA)	Stool samples were collected at 1 year after discharge	Alpha diversity decreased in long COVID patients compared to COVID-19-recovered patients who did not develop long COVID and uninfected controls The compositional structure of the gut microbiota was altered in COVID-19 patients who developed long COVID, as the relative abundance of the bacterial genera *Eubacterium*, *Agathobacter*, *Subdoligranulum*, *Ruminococcus*, and *Veillonella* was significantly different in these subjects compared to COVID-19 patients who did not develop long COVID and uninfected controls
[59]	June 2023	N = 18 (14 SARS-CoV-2+ cases and 4 uninfected controls) Relevant features: all the infected subjects enrolled in this study had mild COVID-19 disease and remained outpatients, whereas uninfected individuals were household controls	16S rRNA gene sequencing (V4) Sequencing platform: Illumina NextSeq 550 (Illumina, CA, USA)	Highly heterogeneous. Maximum of 154 days after SARS-CoV-2 initial infection	SARS-CoV-2 infection resulted in a long-lasting disruption and instability of the gut microbial ecology (with the Firmicutes and Actinobacteriota phyla and the *Rothia* genus as the most variable bacterial taxa following SARS-CoV-2 infection)
